# Intracardiac hemodynamic forces using 4D flow: a new reproducible method applied to healthy controls, elite athletes and heart failure patients

**DOI:** 10.1186/1532-429X-18-S1-Q61

**Published:** 2016-01-27

**Authors:** Johannes Töger, Per M Arvidsson, Mikael Kanski, Katarina Steding-Ehrenborg, Gianni Pedrizzetti, Marcus Carlsson, Håkan Arheden, Einar Heiberg

**Affiliations:** 1 Department of Clinical Physiology, Lund University and Lund University Hospital, Lund, Sweden; 2 Physiotherapy, Department of Health Sciences, Lund University, Lund, Sweden; 3 Department of Engineering and Architecture, University of Trieste, Trieste, Italy; 4 Department of Biomedical Engineering, Lund University, Faculty of Engineering, Lund, Sweden

## Background

Blood flow in the left ventricle (LV) is closely linked to the function of valves, great vessels and the myocardium. Previous studies have used the Pressure Poisson Equation (PPE) to compute relative pressure fields from 4D flow data. However, the PPE may be numerically sensitive to errors in velocities and delineations. Hemodynamic forces is a quantitative measure similar to relative pressure maps, which may be less sensitive to errors.

Therefore, the aim of this study was to investigate the reproducibility of hemodynamic force quantification, and to present initial observations in controls, elite endurance athletes and patients with heart failure.

## Methods

We included 38 healthy volunteers (24 controls and 14 elite endurance athletes) and 10 patients with heart failure. Cardiovascular magnetic resonance at 1.5T or 3T was performed on all subjects, including cine long-axis and short-axis images covering the LV, and 4D flow (retrospectively ECG-gated, voxel size 3 × 3 × 3 mm, temporal resolution 50 ms). Subsets of the controls were scanned on 1.5T and 3T scanners (n = 6) or with and without respiratory navigator gating (Resp+ and Resp-) for the 4D flow (n = 8). Furthermore, the effect of LV delineation was investigated by comparing manual and automatic segmentations [1] (n = 21). Reproducibility was expressed as mean ± 2SD of differences.

The intraventricular pressure gradient was calculated from 4D flow data using the Navier-Stokes equations and was integrated over the LV to produce the hemodynamic force normalized for LV volume (units: mN/ml). The hemodynamic force was decomposed into septal-lateral, inferior-anterior and basal-apical components. The temporal root mean square (RMS) force was computed for systole and diastole separately.

## Results

Good reproducibility was found for 1.5T vs 3T (y = 0.84 × +0.07, R^2^ = 0.86, bias -0.02 ± 0.34 mN/ml), Resp+ vs Resp- (y = 1.01 × +0.01, R^2^ = 0.85, bias -0.01 ± 0.37 mN/ml) and automatic vs manual segmentation (y = 0.91 × +0.03, R^2^ = 0.90, bias -0.02 ± 0.18 mN/ml).

**Figure 1:**
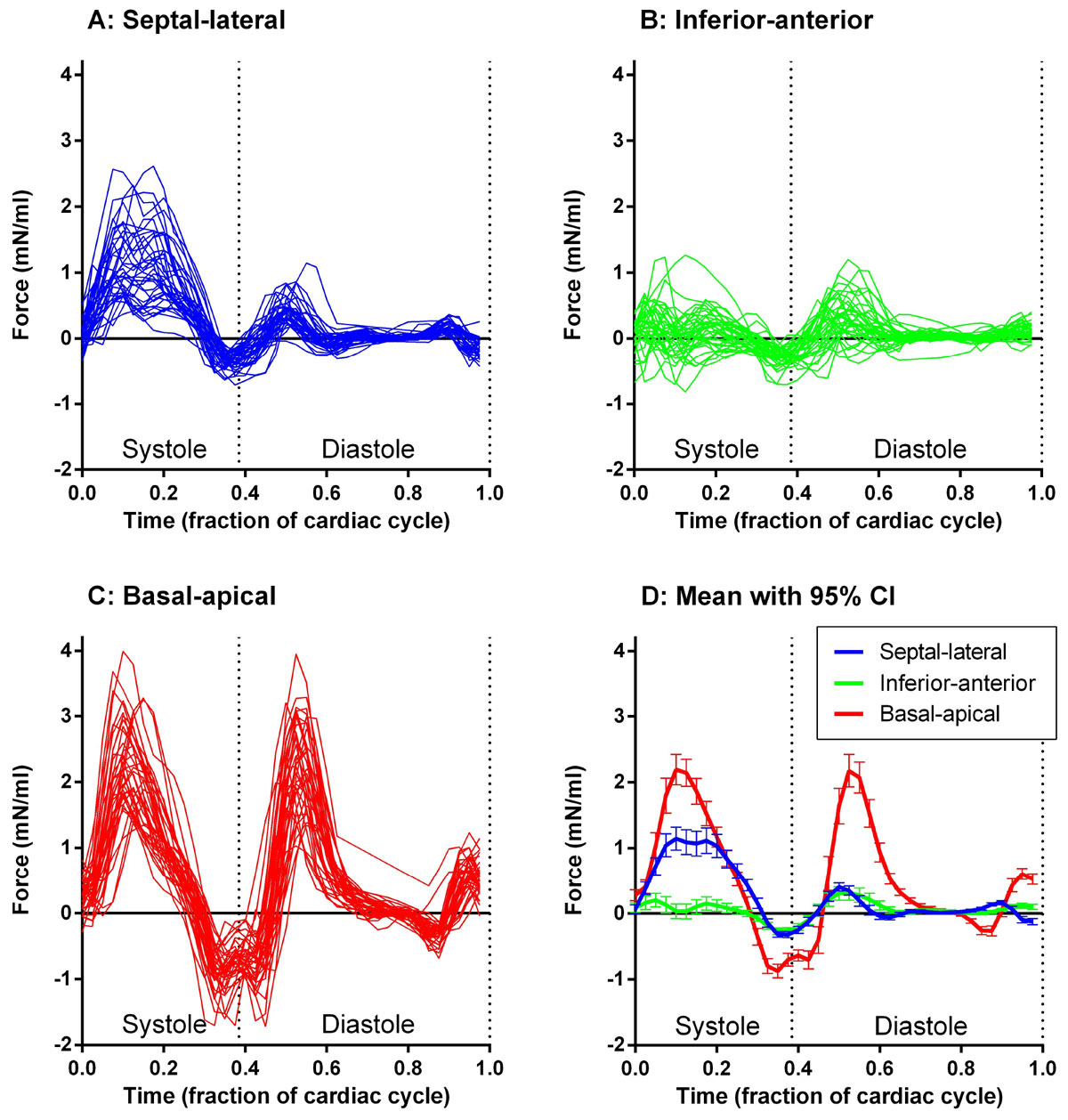
**Hemodynamic force curves in the left ventricle in healthy volunteers (controls and athletes, n = 38)**. Panel A shows the septal-lateral component (blue), Panel B the inferior-anterior component (green) and Panel C the basal-apical component (red). Panel D shows the mean and 95% confidence interval (CI) of the mean for all curves. Individual force curves have been resampled to a reference heart beat.

**Figure 2:**
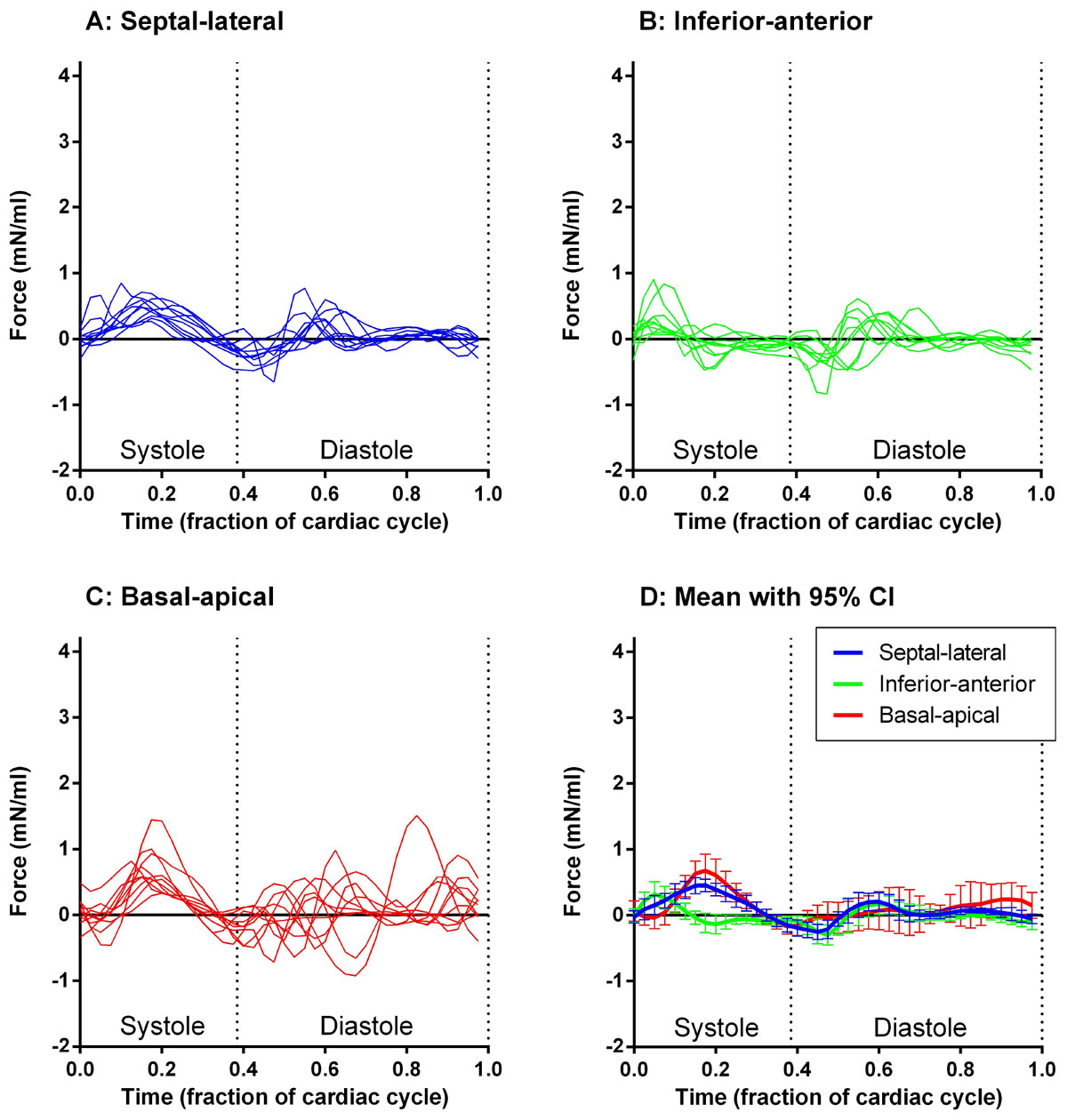
**Hemodynamic force curves in the left ventricle in patients (n = 10)**. Panel A shows the septal-lateral component (blue), Panel B the inferior-anterior component (green) and Panel C the basal-apical component (red). Panel D shows the mean and 95% confidence interval (CI) of the mean for all curves. Individual force curves have been resampled to a reference heart beat.

Hemodynamic force curves are shown for controls and athletes in Figure 1 and for patients in Figure 2.

In systole, RMS hemodynamic forces were larger in healthy volunteers than in patients (septal-lateral: 0.81 ± 0.34 vs 0.30 ± 0.08 mN/ml, p < 0.0001, inferior-anterior: 0.27 ± 0.13 vs 0.19 ± 0.10, p = 0.022, basal-apical: 1.30 ± 0.36 vs 0.37 ± 0.15, p < 0.0001). In diastole, only the basal-apical component was larger in healthy volunteers than in patients (septal-lateral: 0.21 ± 0.09 vs 0.17 ± 0.09, p = 0.26, inferior-anterior: 0.21 ± 0.09 vs 0.20 ± 0.07, p = 0.86, basal-apical: 0.94 ± 0.25 vs 0.34 ± 0.18, p < 0.0001).

## Conclusions

Hemodynamic forces computed from 4D flow MR gives a reproducible measure of blood flow dynamics. The observed differences between healthy volunteers and patients may be used to increase our understanding of the physiology and pathophysiology of the heart.

